# Data of *de novo* assembly and functional annotation of the leaf transcriptome of *Impatiens balsamina*

**DOI:** 10.1016/j.dib.2018.12.042

**Published:** 2018-12-16

**Authors:** Lian Chee Foong, Anthony Siong Hock Ho, Brandon Pei Hui Yeo, Yang Mooi Lim, Sheh May Tam

**Affiliations:** a School of Biosciences, Faculty of Health and Medical Sciences, Taylor’s University, Jalan Taylors, 47500 Subang Jaya, Selangor, Malaysia; b Formerly of the Faculty of Engineering Computing and Science, Swinburne University of Technology Sarawak Campus, Jalan Simpang Tiga, 93330 Kuching, Malaysia; c Department of Pre-Clinical Sciences, Faculty of Medicine and Health Sciences, Universiti Tunku Abdul Rahman, Lot PT 21144, Jalan Sungai Long, Bandar Sungai Long, 43000 Kajang, Selangor, Malaysia

## Abstract

*Impatiens balsamina* is both an ornamental and pharmacologically important plant widely distributed in many Asian countries. The leaf of the plant contains many secondary metabolites possessing anti-microbial, anti-tumour and anti-cancer properties. Though there are many phytochemical studies done on the different natural extracts for this plant, not much of genetic information is currently available. This is the first transcriptome of *I. balsamina* leaf using paired-end Illumina HiSeq sequencing which generated 10.79 GB of raw data. Information of pre-processing (reads filtering), *de novo* assembly and functional annotation are presented. This data is accessible via NCBI BioProject (PRJNA505711).

## Specifications table

**Table:** 

Subject area	Plant Science
More specific subject area	Transcriptomics
Type of data	Table, figure
How data was acquired	Illumina HiSeq. 2000 sequencing platform
Data format	Raw, analysed
Experimental factors	Total RNA was isolated from the leaves of *I. balsamina*
Experimental features	Total RNA of the pink, multi-petal *I. balsamina* was extracted from the leaves and used for cDNA library construction, followed by generating paired-end sequencing data using Illumina HiSeq. 2000 system. After pre-processing of the raw reads, Trinity software was used to perform *de novo* assembly of 9.97 GB clean reads. Functional annotation using BLASTx searches against several online databases including NCBI non-redundant (Nr) protein database, Swiss-Prot, Pfam, InterPro, PROSITE protein databases, as well as Kyoto Encyclopedia of Genes and Genomes (KEGG), and Gene Ontology (GO).
Data source location	Subang Jaya, Selangor, Malaysia (3.0643° N, 101.6174° E)
Data accessibility	Data is with this article and the raw sequence data has been deposited in the SRA database (PRJNA505711).
Related research article	Lian Chee F, Jian Yi C, Anthony Siong Hock H, Brandon Pei Hui Yeo, Yang Mooi L, Sheh May T. RP-HPLC quantification of naphthoquinones (lawsone and MNQ) and transcriptomics study of genes and pathways involved in naphthoquinones biosynthesis in *Impatiens balsamina* (Balsaminacae). Plant Gene, “under review”.

## Value of the data

• This is the first *de novo* leaf transcriptome that significantly increased amount of sequence information available for this plant, also useful as reference to other Impatiens species.
• The annotated transcripts against KEGG pathways could be useful for researchers working on detailed protein-coding genes of this plant related to transcripts, genes and pathways involved in biosynthesis of secondary metabolites.
• This data will serve as a useful transcriptomic resource for future studies including gene expression, RNAi induction analysis, genomics and functional genomics in *I. balsamina* and other *Impatiens* species.

## 1 Data

**Fig. 1:**
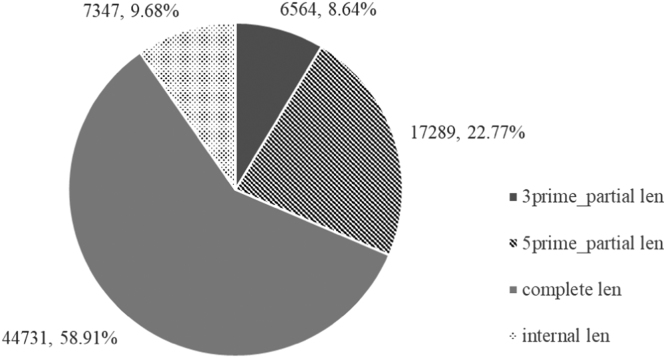
Types of coding sequences (CDS, with a minimum of 100 amino acids in length) predicted from the assembled leaf transcripts of *Impatiens balsamina.* Complete len = presence of start and stop codons in the open reading frames (ORF) of the CDS; 5 prime_partial len = missing stop codon the ORF; 3 prime_partial len = missing start codon in the ORF; Internal len = neither start nor stop codons were detected in the ORF.

Here, we present the first leaf transcriptomic data of *I. balsamina* generated using Illumina HiSeq. 2000 sequencing technology. The sequencing run generated a total of 10.79 GB (106,867,578 reads) raw data in FASTQ format (has been deposited in the SRA database; PRJNA505711). After pre-processing, *de novo* assemble of the clean reads (9.97 GB, 99,258,630 reads) was performed and the information was summarised in Table 1. The analysis showed that 82.65 % of the total transcripts (75,931 sequences) contained putative coding sequences (CDS). Among the CDS, 58.91 % of CDS had a complete open reading frame which containing defined start and stop codons (Fig. 1). Other than that, 31,200 transcripts were classified as partial CDS. Specifically, 17,289 transcripts were classified as “5 prime_partial len” containing a stop codon and missing start codon, 6564 were grouped as “3 prime_partial len” containing a start codon and lacking stop codon, and 7347 were categorised as “internal len” with missing of both the start and stop codons. A total of 2253 KO IDs was assigned to 24,988 CDS and mapped to 387 KEGG pathways. The output of pathway mapping using KAAS is presented in Supplementary material S1. Table 2 shows an overview of the bioinformatic tools used to analyse the leaf transcriptome of *I. balsamina*.

**Table 1:** Assembly statistics for the leaf transcriptome of *Impatiens balsamina*.

**Summary**	**Assembled transcripts**
**Total assembled transcripts**	91,873
**Total assembled unigenes**	66,294
**Percent GC**	42
**N50 contig length**	1544
**Average assembled transcripts (nt)**	943
**Total assembled reads (nt)**	86,600,995

**Table 2:** Bioinformatic tools used in the analysis of the leaf transcriptome of *Impatiens balsamina*.

Software/program	Website	Ref.
FastQC v0.11.5	https://www.bioinformatics.babraham.ac.uk/projects/fastqc/	[2]
Trimmomatic v.0.35	http://www.usadellab.org/cms/?page=trimmomatic	[3]
Prinseq v0.20.4	http://prinseq.sourceforge.net/	[4]
Trinity	https://github.com/trinityrnaseq/trinityrnaseq/wiki	[10]
Bowtie v1.1.2	http://bowtie-bio.sourceforge.net/index.shtml	[6]
RSEM v.1.2.11	https://github.com/deweylab/RSEM	[7]
BLAST2GO	https://www.blast2go.com/	[11]
TransDecoder	https://github.com/TransDecoder/TransDecoder/wiki	[8]
KAAS ver. 1.6	http://www.genome.jp/tools/kaas/	[9]
MEGA 7.0.26	https://www.megasoftware.net/	[12]

## 2 Experimental design, materials, and methods

### 2.1 Plant material

Cultivated plants of *I. balsamina* (pink, multi-petal form) were obtained from local nursery located in Selangor, Malaysia. The plants were then continuously seed-propagated at the plant growth area, Taylor’s University. To minimize sampling variation, leaves were collected and pooled from six plants (age three-months-old). Leaf samples were immediately frozen in liquid nitrogen upon harvesting.

### 2.2 Total RNA extraction, cDNA library construction and transcriptome sequencing

Total RNA extraction was conducted in triplicates according to an optimized protocol described in [1]. One sample of high-quality intact RNA (RIN > 7.5; *A*_260_/*A*_280_ and *A*_260_/*A*_230_ ratios > 1.9) was then selected for sequencing. Two μg of total RNA from the sample was used in the mRNA-seq library construction. The mRNA was isolated and fragmented to 200 nt for cDNA synthesis. The cDNA was end-repaired, ligated to adapters and PCR-enriched using the NEB Next Ultra RNA Library Prep Kit for Illumina (NEB, USA) according to the manufacturer’s protocol. The final library was quantified using a Qubit DNA HS assay and library size determined using a Bioanalyzer High Sensitivity DNA chip. Sequencing of the final library was performed by Malaysian Genomics Resource Centre Berhad (MGRC) using the Illumina HiSeq. 2000 (Illumina, USA) platform. Paired-end sequencing was performed at 2 × 100 bp per cycle, with 200 cycles.

### 2.3 Sequence data assembly and bioinformatic analysis

Quality assessment of the reads was performed using FASTQC v0.11.5 [2]. Raw reads were pre-processed using Trimmomatic (version 0.35) [3] and Prinseq (version 0.20.4) [4] to remove the adapter sequences and low quality reads with ambiguous base (N). The sequences with length below 75 were discarded and the remaining sequences were named as ‘clean reads’. Trinity software (version 2.2.0) with default parameters was used to *de novo* assemble the clean reads. Transcript and gene IDs were assigned to clean reads according to the default criteria determined by Trinity.

A bioinformatic analysis was performed using the clean reads and the results were reported in [5]. Briefly, bowtie (version 1.1.2) [6] was used to assess the quality of assembled transcripts by mapping the clean reads against assembled transcripts. RSEM (version 1.2.11) software package [7] was used to estimate the abundance of transcripts/unigenes. Functional annotations were accomplished by performing BLASTx searches with an E-value threshold of ≤ 1e^-5^ against the NCBI non-redundance protein database, limited to ‘green plant (txid 33090)’, as well as other established databases such as Swiss-Prot-, Pfam-, InterPro- and PROSITE protein databases. Finally, BLAST2GO program was used to assign gene ontology (GO) terms (*E*-value ≤ 1e^-5^) to categorise the transcripts.

TransDecoder [8] was used to identify coding sequences (CDS) with open reading frames (ORFs) of at least 100 amino acids in length from the assembled transcripts. Kyoto Encyclopedia of Genes and Genomes (KEGG) Ortholog (KO) assignment and mapping of amino acid sequences to biosynthesis pathways were performed using KEGG automatic annotation server (KAAS; version 1.6) [9], with default threshold bit-score value of 60, single-directional best hit (SBH) method, BLASTx program, and the selected KEGG database included 32 eudicots, two monocots and one basal magnoliophyte. Pathway mapping analysis from KAAS is presented in Supplementary material S1.

## Appendix A Supplementary material

Supplementary material Table S1. Results of KAAS pathway mapping of the translated transcripts (coding sequences with minimum 100 amino acids) of *Impatiens balsamina*.
