# Supplementary material for: Data of de novo assembly and functional annotation of the leaf transcriptome of Impatiens balsamina
Source: Data Brief. 2018 Dec 16;23:103603. doi: 10.1016/j.dib.2018.12.042 (PMC6378892; doi:10.1016/j.dib.2018.12.042)
Supplement: Supplementary file 1 — Supplementary material [file mmc1.docx]

**Conflict of Interest**

December 11, 2018

Editor-in-Chief, Data in Brief

Dear Sir,

We would like to submit this author declaration letter for the revised manuscript (DIB-D-18-03063R1). We wish to confirm that there are no known conflicts of interest associated with this publication and there has been no significant financial support for this work that could have influenced its outcome.

We confirm that this work is original and has not been published elsewhere nor it is currently under consideration for publication elsewhere. Besides that, the manuscript has been read and approved by all named authors and there are no other persons who satisfied the criteria for authorship but are not listed. We also confirm that the order of the authors listed in the manuscript has been approved by all of us.

We confirm that we have given due consideration to the protection of intellectual property associated with this work and that there are no impediments to publication, including the timing of publication, with respect to intellectual property. In so doing we confirm that we have followed the regulations of our institutions concerning intellectual property.

We understand that the Corresponding Author (Sheh May Tam) is the sole contact for the Editorial process (including Editorial Manager and direct communications with the office). She is responsible for communicating with the other authors about progress, submissions of revisions and final approval of proofs. We confirm that we have provided a current, correct email address which is accessible by the Corresponding Author and which has been configured to accept email from [shehmay.tam@taylors.edu.my](mailto:shehmay.tam@taylors.edu.my).

P/S: Please note that there is no download link for Conflict of Interest form from Data in Brief’s portal, and thus we have manually written this letter

Signed by all authors as follows:

Lian Chee Foong

Anthony Siong Hock Ho

Brandon Pei Hui Yeo

Yang Mooi Lim

Sheh May Tam
